# Full-length cloning, sequence analysis and expression detection of the β*-*tubulin gene from the Chinese gall aphid (*Schlechtendalia chinensis*)

**DOI:** 10.1038/s41598-017-06806-8

**Published:** 2017-07-25

**Authors:** Ping Liu, Zi-Xiang Yang, Xiao-Ming Chen, Hang Chen

**Affiliations:** 1 Research Institute of Resource Insects, Chinese Academy of Forestry, Key Laboratory of Breeding and Utilization of Resource Insects of State Forestry Administration, Kunming, Yunnan China; 2 Yunnan Forestry Technological College, Kunming, Yunnan China

## Abstract

Some insect galls are formed on sumac plants by certain aphid species and have been used for medicinal and chemical purposes as they are rich in tannins. The most prominent species among gall aphids in China is *Schlechtendalia chinensis*, which formed horn-shaped galls on the winged rachis of *Rhus chinensis*. *S. chinensis* has a complex life cycle, with a switch of hosts between *R. chinensis* and certain mosses, and a switch of sexual and asexual reproduction (cyclical parthenogenesis). We have cloned a full-length cDNA of the β*-*tubulin gene from *S. chinensis*, using qPCR and RACE. This cDNA has 1606 base pairs with a 251 bp 5′-untranslated region (5′-UTR) and a 15 bp 3′-untranslated region (3′-UTR). The gene encodes a protein with 376 amino acids residues. The expression levels of the β*-*tubulin gene in *S. chinensis* were investigated among fundatrigeniae and overwintering larvae rearing under either natural conditions, or at 7.5 °C and 18 °C. No significant differences (P > 0.01) in gene expression levels were found in insects under these conditions. It is indicates that the β-tubulin gene is highly conserved and then it may be used as a reference for further research in gene expression and reproduction determination in this important aphid.

## Introduction

Certain aphid galls are structures of plant tissues induced by aphids on plants in the tribe Fordini (Hemiptera, Aphididae, Eriosomatinae). There are eleven species of aphids (in six genera) that can form galls on *Rhus* trees commonly found in eastern Asia, especially in southwest China. These galls (also called Chinese Gall) have historically been used for medicinal and chemical purposes, as they are rich in tannins, which accounting for about 50–75% of total gall dry weight. They are commonly used as a source of tannic, gallic, and pyrogallic acids^1^.

The gall aphid *Schlechtendalia chinensis* is the main species among the gall aphids in China. The aphid induces horned-galls on the sumac, *R. chinensis* (Anacardiaceae), and accounting for more than 70% of Chinese gallnuts annually. The life cycle of *S. chinensis* includes sexual and asexual reproduction and a host switch between *R. chinensis* and certain mosses (Mniaceae). In the early spring, the winged morph (sexupara) migrates from moss to *R. chinensis* and produces male and female offspring (sexuales). Each mated female produces a fundatrix ovoviparously, which then moves toward and feeds on the new foliage, where it initiates gall formation. Usually each gall is induced by a single fundatrix and her offspring (the fundatrix reproduces parthenogenetically for three generations of fundatrigeniae within a gall). When the horned-gall matures and dehisces in autumn, the alate fundatrigenia migrates from the gall to its secondary host-plant, mosses. Then it asexually produces larvae that live in the tender stem of mosses and excrete wax to develop a protective sheet for the winter. In the following spring, the larvae moult and form alate sexuparae, which then fly back to *R. chinensis* and begin the next life cycle^2–4^. The occurrence of cyclical parthenogenesis (sexual and asexual reproduction alternation) depends on temperature, photoperiod, and host plants^5–11^.

There are seven members of the tubulin family that have been identified, including α*-*, β*-*, γ*-*, δ*-*, ε*-*, ζ*-*, and η*-*tubulin in various organisms. Genes coding for α*-*, β*-*, and γ*-*tubulins are found in all eukaryotes^12^. The *α-* and β*-*tubulin are the main types of related proteins, forming a heterodimer that is the major building block of microtubules. Tubulins have cell-type specificity in different developmental stages and tissues, performing different physiological functions, and are indispensable proteins maintaining cell shape, movement and intracellular transport of substances^13–16^. They are essential components of the cytoskeleton and spindles. It has been suggested that tubulins also play an important role in oocyte meiosis and cell mitosis^17, 18^. In recent years, a growing number of reports have suggested that differentially expressed genes during different stages or in different tissues regulate the growth, development and aging of an organism^17, 19^. Tubulins are expressed differently in developmental stages of some insects, usually with higher levels of expression in the parthenogenetic cycle^20, 21^. *S. chinensis* has a complex life cycle, with both parthenogenetic and sexual generations, and the levels of tubulin expression during these two types of reproductions are unclear. In this study, we cloned a β*-*tubulin gene from *S. chinensis*. The cloning of β*-*tubulin gene from this economically important, genomically understudied aphid is useful to better understand its structure, function, and expression in the different modes of reproduction. The availability of this gene provides a useful control during expression analysis of other genes in *S. chinensis*.

## Results

### Nucleic acids quality

**Figure 1:**
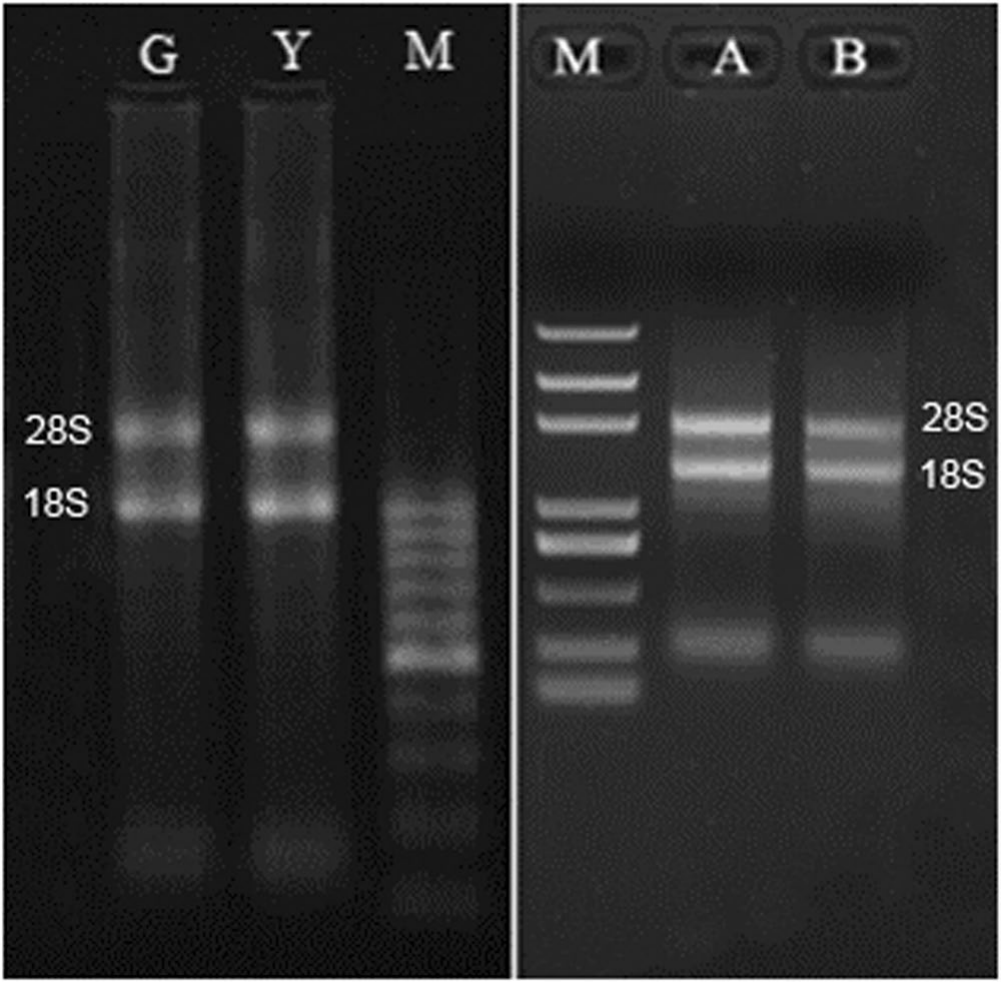
Total RNA bands on the gel by electrophoresis. M: Marker. G: fundatrigenia. Y: overwintering aphid. A: overwintering aphid reared at constant 18 °C. B: overwintering aphid reared at constant 7.5 °C.

After the extraction of total RNA, the quality of the nucleic acids was tested by spectrophotometry. And the integrity of RNA samples was showed that 28 S and 18 S bands were clear on 1% agarose gels after electrophoresis (Fig. 1). The ratios of OD260/OD280 equal or above 2.0 indicated that the purity of RNA samples meets requirements.

### 5′ RACE and 3′ RACE

**Figure 2:**
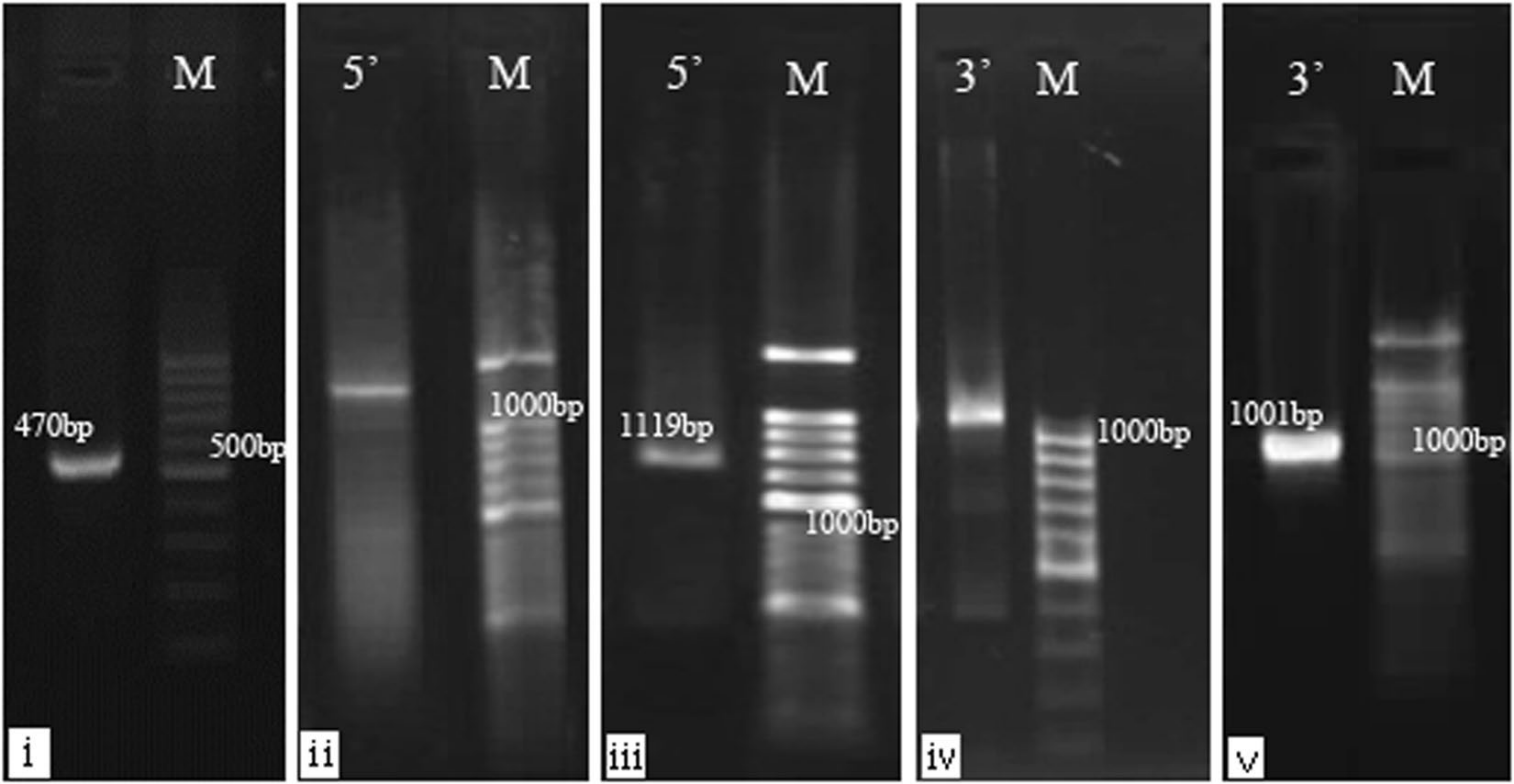
Gel electrophoresis bands of PCR products.

Using the P1/P2 primer pair, a cDNA fragment of 470 bp was obtained and sequenced (Fig. 2i). To obtain the 5′-uncovered region, a new specific primer, P4, was synthesized based on the cloned fragment. The P4 specific primer together with the universal adapt primer were used for the first-round PCR amplification during 5′-RACE and the banding pattern of the PCR products is shown in Fig. 2ii. A second round of nested PCR amplification with the P3 and the adapt primers yielded a single band with size around 1 kB (Fig. 2iii).

To clone the 3′-uncovered region, the P6 and oligo-dT primers were used for the first round PCR amplification and defused bands were observed on an agarose gel (Fig. 2iv). A second round of nested PCR with the P5 and oligo-dT primers yielded a clear band, again around 1 kB (Fig. 2v).

### Open reading frames analysis of the tubulin gene

**Figure 3:**
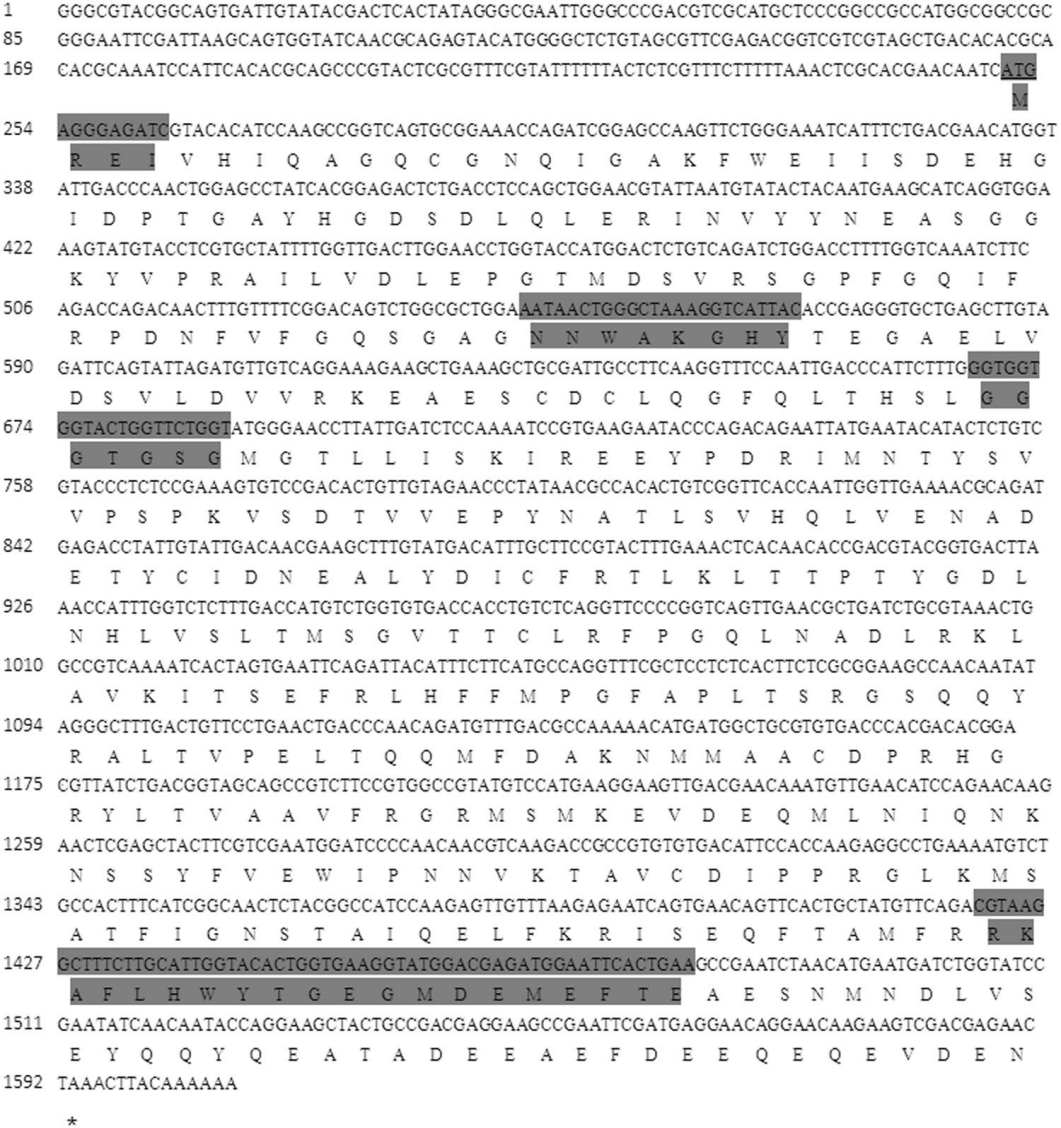
Nucleotide and deduced amino acid sequences of the cloned tubulin gene.

The size of the cloned tubulin transcript was 1606 bp. The initiation codon ATG was located at the site 251, and termination codon at the site 1592. This open reading frame (ORF) encodes a protein with 447 amino acids. It has a 251 bp 5′-UTR and a 15 bp 3′-UTR. Amino acid sequence analysis showed that the β*-*tubulin amino acid sequence contained two conservative sequences (NNWAKGHY and RKAFLHWYTGEGMDEMEFTE), a GTP binding site (GGGTGSG), and a post-transcriptional control signal MERI (Fig. 3).

### Homology analysis

**Figure 4:**
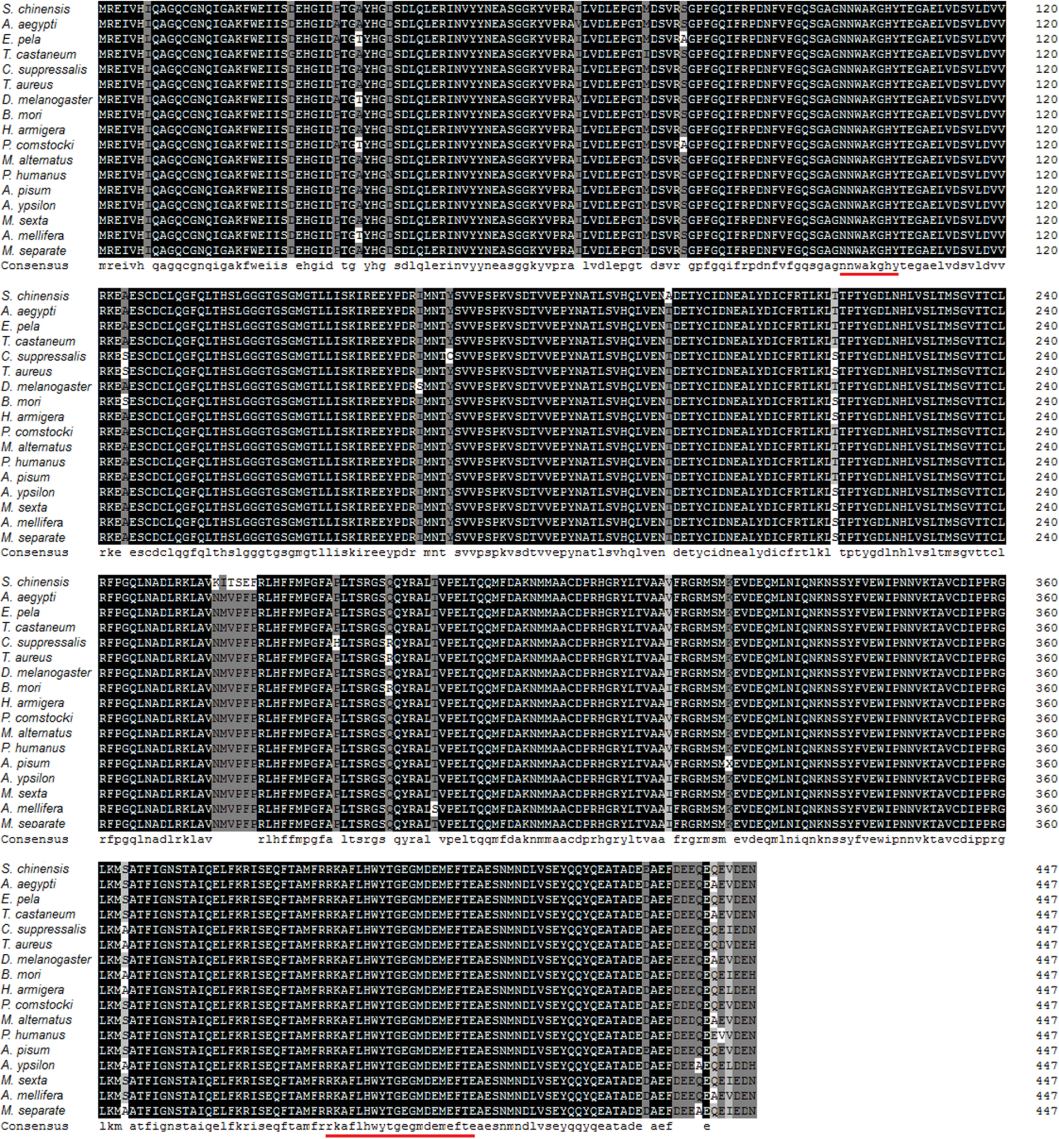
Amino acid sequence alignment of the predicted *S. chinensis* β-tubulin with homologs from other insect species.

The β*-*tubulin amino acid sequence we obtained shared >95% similarity with proteins from the NCBI database. The highest sequence similarity was with tubulins from pea aphid (*Acyrthosiphon pisum*), red flour beetle (*Tribolium castaneum*), and long-horned beetle (*Monochamus alternatus*). An alignment of the β*-*tubulin amino acid sequence of *S. chinensis* with those from 16 other insect species is shown in Fig. 4.

### Structure prediction

The predicted β-tubulin protein has a relative molecular weight of 50.22 kDa, and an isoelectric point of 4.77. Proscan analysis indicated that the β*-*tubulin protein has many potential protein modification sites, such as three N-glycosylation sites (184–187, 337–340, 370–373), binding sites for cAMP and cGMP (379–382), five phosphorylation motif sites for protein kinase c (75–77, 172–174, 214–216, 274–276, 322–324), seven phosphorylation sites for casein kinase II (115–118, 178–181, 221–224, 285–288, 322–325, 409–412, 429–432), and 16 N-myristoylation sites (10–15, 13–18, 29–34, 34–39, 71–76, 93–98, 96–101, 98–103, 140–145, 141–146, 142–147, 144–149, 235–240, 244–249, 360–365, 369–374).

**Figure 5:**
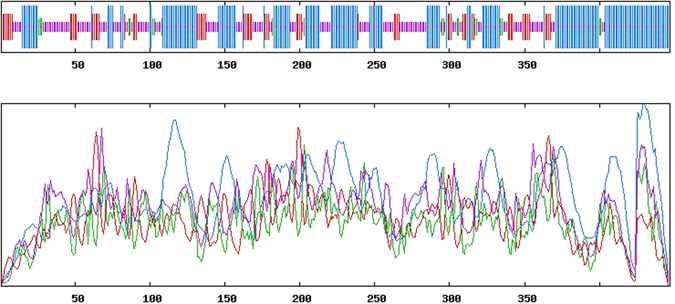
The predicted secondary structure of β*-*tubulin in *S. chinensis*. Red lines represent the conserved domain of β-tubulin amino acids.

Secondary structure prediction suggested that the protein has a relatively large amount of beta-sheet. Specifically, the predicted structure comprised of 45.9% alpha helix, 34.9% coil, 4.5% β-turn, while the remaining 14.8% is chain extension (Fig. 5). No signal peptide was found based on SignalP analysis, suggesting that the β-tubulin protein is a non-secreted protein.

### Expression analysis

**Figure 6:**
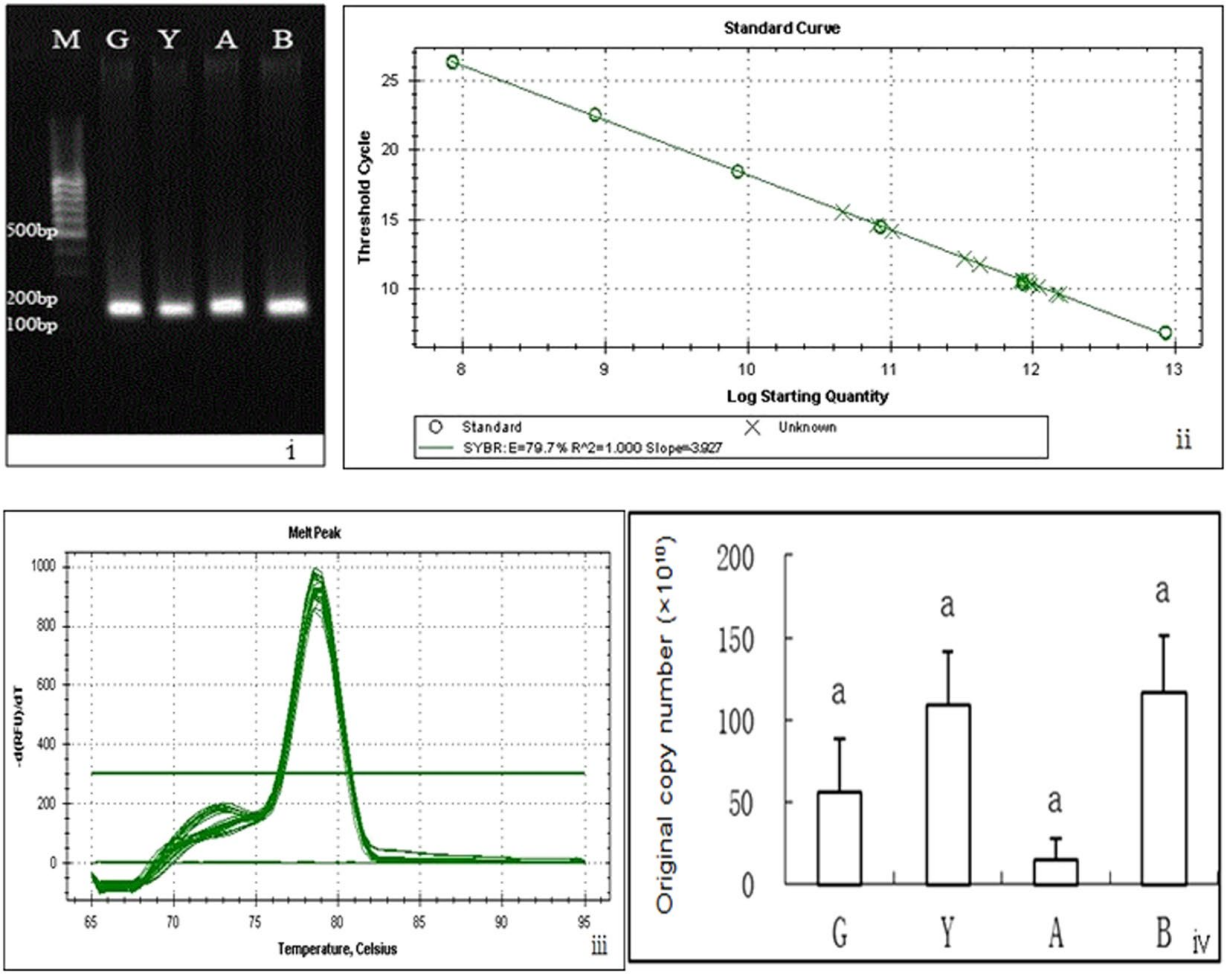
Expression assays of the tubulin gene among different phases of *S. chinensis*. (i) Gel electrophoresis of amplified product. (ii) Standard curve. (iii) Melt peak of tubulin gene. (iv) Quantitative assays of expression level: P > 0.01. M: Marker. G: fundatrigenia. Y: overwintering larvae reared at natural condition. A: overwintering larvae reared at constant temperature 18 °C. B: overwintering larvae reared at a constant temperature 7.5 °C. Same letter on the bar of the bar graph mean no significant difference.

RNA from four developmental phases of *S. chinensis* was respectively isolated for analysis of expression levels of β*-*tubulin. Condition for PCR amplification was optimized so that a single band was present in each PCR reaction (Fig. 6i), and that a positive linear relationship existed between the concentrations of standard substance and fluorescence signal (Fig. 6ii). In qPCR analysis, we found a single melt peak was observed, suggesting high specificity of the primers and a unique PCR product (Fig. 6iii). The expression level of the β*-*tubulin gene varied a little among the fundatrigeniae, and overwintering larvae reared either under natural conditions, or at high-temperature 18 °C, and low-temperature 7.5 °C, with CT values 5.64 × 1011 copies/ μl, 1.09 × 1012 copies/μl, 1.44 × 1011 copies/μl and 1.17 × 1012 copies/μl, respectively. However, there were no significant differences at P > 0.01 among different samples based on statistical analysis (Fig. 6iv).

## Discussion

In this study, we cloned a full-length cDNA of a *S. chinensis* β*-*tubulin gene by PCR and RACE. The transcript contained 1606 bp. Amino acid sequences of the predicted protein shares greater than 95% similarity to the homolog of the pea aphid, suggesting that the β*-*tubulin gene is highly conserved during evolution. Proscan analysis revealed the β*-*tubulin protein contains many potential protein modification sites.

Tubulins are important components of the cytoskeleton and spindle. It also plays essential roles in cell division and the start of mitosis in oocytes^17^. Tubulins also participate in important physiological activities such as cyclosis, form maintenance, cell division and differentiation, signal transduction, and polarity construction^14–16^. The oocyte of the parthenogenetic viviparous pea aphid can self-organize microtubule-based asters, which in turn interact with the female chromatin to form the first mitotic spindle^18^. Yang^20^ and Yu^21^ reported that the expressions of tubulin genes are different between sexual and asexual reproduction modes. Expression level is higher in parthenogenetic phase than its bisexual reproduction phase in snout beetle (*Lissorhoptrus oryzophilus*). We did see variation in expression levels of the β*-*tubulin gene in the four reproductive/life stage phases of *S. chinensis*. However, the variations among different phases of this aphid were not statistically significant with P > 0.01. This observation suggests that the β*-*tubulin protein is highly conserved during evolution and therefore is expressed similarly in different developmental stages of *S. chinensis*. Because of this characteristic, it may be used as a reference gene for further genetic research.

The life cycle of *S. chinensis* is complex, showing not only reproductive alternation but also telescoping of generations, where the mature embryos developing inside the maternal abdomen carry the first developmental stage of the third generation. When environmental factors change, such as photoperiod and temperature, the parental generation may also be affected. Therefore, the embryonic development and phenotype of offspring, such as alate or apterous and sexual vs. parthenogenic reproduction, may be affected as well^22–24^. Since the sexuale and sexupara of *S. chinensis* are predetermined at overwintering larvae (telescoping of generations)^25^, the β-tubulin gene expression in overwintering larvae would not be linked with either parthenogenesis or bisexual reproduction. *S. chinensis* has a complex life cycle, alternating between sexual and asexual generations, and also displays polymorphism and host plant alternation. These characteristics make molecular studies more difficult compared with other insects. Despite the huge economic importance of *S. chinensis*, this insect species remains as an understudied organism molecularly. The cloning of the β*-*tubulin gene may provide a reference for further research on gene expression in *S. chinensis* and other aphids.

## Materials and Methods

### Aphids from natural conditions

The wingless fundatrigenia of aphid, *S. chinensis* were collected from the horned galls in mid-August and homogenized immediately in Trizol and then stored at −70 °C until being analyzed.

The secondary host plant moss, Plagiomnium maximoviczii was planted in plastic trays which covered with 2 cm sandy loam soil in August at Emei, Sichuan province, China. All trays with the moss were put in the field through conventional management. In October, when the horned galls matured and dehisced, they were harvested and placed next the trays. The alate fundatrigeniae migrated from the dehisced galls to the trays nearby and produced larvae asexually which fed on the tender moss stems. In the following spring, the overwintering larvae were collected from the mosses and homogenized immediately in Trizol and then stored at −70 °C until being analyzed.

### Overwintering aphids under artificial conditions

In October, parts of the trays above were moved to an incubator and cultured under 75% relative humidity, a 13:11 h L:D photoperiod and constant 7.5 °C or 18 °C, respectively. In the following spring, the overwintering larvae were collected from the mosses and homogenized immediately in Trizol and then stored at −70 °C until being analyzed.

### RNA extraction

Total RNA was extracted using a Trizol kit (Invitrogen, CA, USA) following the manufacturer’s instruction. When the aphid tissues were dissolved and homogenized, the samples were centrifuged at 14,000 × gravity (g) for 5 min at 4 °C. After discarding the precipitates, chloroform was added to the solution at the ratio of 200 μl of chloroform per 1 ml Trizol. The tubes were then mixed vigorously for 15 seconds, and followed by incubation at room temperature for 15 minutes. The samples were then centrifuged at 14,000 × g for 15 minutes at 4 °C. Then ~500 μl of the colorless, upper phase solution containing RNA was transferred to a fresh RNase-free tube. Cymene was then added at 1:1 ratio. After vortexing and incubation at room temperature for 10 minutes, the sample was then centrifuged at 14,000 × g for 10 minutes at 4 °C. The supernatant was discarded and 1 ml of 75% ethanol per 1 ml Trizol was added. Finally, the sample was centrifuged at 14,000 × g again for 5 minutes at 4 °C and the supernatant was then decanted. The extracted RNA was dried at room temperature and then dissolved in 20 μl of RNase-free water.

### Primer design and synthesis

**Figure 7:**
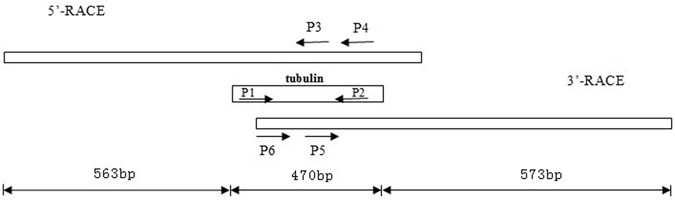
Principle of primer designed for coloning the β*-*tubulin gene from *S. chinensis*.

Primers were designed according to the sequences of β*-*tubulin gene from *Ericerus pela* (GenBank Accession JF731244.1) and *Maconellicoccus hirsutus* (GenBank Accession EF070480.1) using the Primer 5.0 and DNAman software. The directions and primers-covering regions are shown in Fig. 7 and primer sequences are shown in Table 1. The P1/P2 pair was used for the initial cloning of the conserved portion of the β-tubulin gene. The specific primers P3 and P4 and P5 and P6 were designed based on the cloning β-tubulin gene fragment and were used for 5p-RACE and 3p-RACE, respectively. All primers were synthesized by Shanghai Sangon (Shanghai, China).

**Table 1:** Primers designed for coloning β*-*tubulin gene from *S. chinensis*.

Primer	Code of primer	Sequence of primer(5′ → 3′)
Tubulin F	P1	TGCGGWAAYCAAATCGGAGC
Tubulin R	P2	CTGAWARSGTRGCRTTGTASG
5′ RACE Outer	P3	CTGTCTGGGTATTCTTCACGGATTT
5′ RACE Inner	P4	TACGGAAGCAAATGTCATACAAAGC
3′ RACE Outer	P5	AGAACCCTATAACGCCACACT
3′ RACE Inner	P6	CCGTGAAGAATACCCAGACAGAAT
Tubulin F	P7	TGTCTGCCACTTTCATCGG
Tubulin R	P8	ATTCCATCTCGTCCATACCTTC

### Tubulin gene cloning and sequencing

Total RNA of *S. chinensis* was used as the template for first strand cDNA synthesis with an oligo-dT as primer using an M-MLV First Stand Kit (Invitrogen, USA). The reaction mixture consisted of 0.5 μl cDNA synthesis mix, 1 μl dNTP, 1 μl Buffer, 0.1 μl rtaq, 0.5 μl P1, 0.5 μl P2, and 6.4 μl H_2_O. After an initial denaturation at 94 °C for 3 min, PCR amplification was carried out for 30 cycles with the following program: 94 °C for 30 s, 53 °C for 30 s, 72 °C for 1 min (30 cycles). The reaction mixture was then incubated at 72 °C for 5 min.

Full length cDNA was obtained using the SMARTER RACE cDNA Amplication Kit (Clontech, USA). Briefly, P4 and the universal primer were used for the first round of PCR amplification and then P3 and the universal primer were used for the second round of PCR amplification during 5′-RACE. Similarly, P6 and the oligo-dT primer were used for the first round of PCR amplification and P5 and the oligo-dT primer were used for the second round of PCR amplification during 3′-RACE. The PCR reaction conditions were the same as described in the previous section.

Recovery, cloning, and sequencing of the DNA fragments were done as follows: The target fragments were purified through electrophoresis on agarose gels and then extracted using a QIAquick Gel Extraction Kit following manufacturer instructions. The target fragments were ligated to a PCR cloning vector with a T4 DNA ligase (Promega, USA) and then transfected into competent cells of DH5-α *Escherichia coli*. Single positive colonies were selected for plasmid DNA isolation for sequencing. Nucleotide sequences were compared and analyzed using NCBI blast and DNAman software.

### Plasmid extraction

Plasmid DNA was extracted from the bacterial cultures using a Plasmid Miniprep Kit (Tiangen, China), and was dissolved in deionized water. DNA concentrations were determined using a spectrophotometer.

### Quantitative real time PCR (qPCR)

qPCR was performed on an ABI 7300 Real-time Detection System (Applied Biosystems). The standard curve of 10^12^ − 10^7^ was drawn according to the concentrations of plasmid DNA. Reaction were carried out in a total volume of 10 μl, containing 1 μl of diluted cDNA mix, 1 μl of each primer (10 mM), 5 μl of iTaq Supermix (Bio-Rad, USA), and 2 μl of Milli-Q water. The reaction procedures was as follows, 94 °C for 3 min, followed 39 cycles with each cycle at 94 °C for 10 s, 53 °C for 20 s, and 72 °C for 30 s. Melting curves were monitored and recorded during each change of temperature, rising from 65 to 95 °C, with three repetitions per sample. To confirm that only one PCR product was amplified and detected, dissociation curve analysis of amplification products was performed at the end of each PCR reaction. After the PCR program, data were analyzed with ABI 7300 SDS software (Applied Biosystems). The comparative CT method was used to analyze the expression levels of the gene.

### Sequences analysis

All sequences were processed using the DNA Star program and compared to other insects species using BLAST (Basic Local Alignment Search Too1) analysis in NCBI (National Center for Biotechnology Information, http://www.ncbi.nlm.nih.gov/). The molecular weight, theoretical pI and amino acid composition parameters were computed by ProtParam tool (http://web.expasy.org/protparam/).
